# The use of mobile phones as a data collection tool: A report from a household survey in South Africa

**DOI:** 10.1186/1472-6947-9-51

**Published:** 2009-12-23

**Authors:** Mark Tomlinson, Wesley Solomon, Yages Singh, Tanya Doherty, Mickey Chopra, Petrida Ijumba, Alexander C Tsai, Debra Jackson

**Affiliations:** 1 Department of Psychology, Stellenbosch University, Private Bag X1, Matieland, 7602, South Africa and Health Systems Research Unit, Medical Research Council, Francie van Zyl Drive, Parrow, Cape Town, 7535, South Africa; 2 Health Systems Research Unit, Medical Research Council, Francie van Zyl Drive, Parrow, Cape Town, 7535, South Africa; 3 Health Systems Research Unit, Medical Research Council, 491 Ridge Road, Durban, 4091, South Africa; 4 Health Systems Research Unit, Medical Research Council, Francie van Zyl Drive, Parrow, Cape Town, 7535, South Africa; 5 School of Public Health, University of the Western Cape, Modderdam Road, Bellville, 7535, South Africa; 6 Chief, Health, UNICEF, United Nations Plaza, New York, NY 10017 USA; 7 Health Systems Research Unit, Medical Research Council, 491 Ridge Road, Durban, 4091, South Africa; 8 Langley Porter Psychiatric Institute, University of California at San Francisco, USA

## Abstract

**Background:**

To investigate the feasibility, the ease of implementation, and the extent to which community health workers with little experience of data collection could be trained and successfully supervised to collect data using mobile phones in a large baseline survey

**Methods:**

A web-based system was developed to allow electronic surveys or questionnaires to be designed on a word processor, sent to, and conducted on standard entry level mobile phones.

**Results:**

The web-based interface permitted comprehensive daily real-time supervision of CHW performance, with no data loss. The system permitted the early detection of data fabrication in combination with real-time quality control and data collector supervision.

**Conclusions:**

The benefits of mobile technology, combined with the improvement that mobile phones offer over PDA's in terms of data loss and uploading difficulties, make mobile phones a feasible method of data collection that needs to be further explored.

## Background

Large field surveys are a common feature of the health research landscape. In low and middle income countries where capacity and administrative problems with the collection of health data are common, surveys are often the only way to collect reliable data [1,2]. Paper based data collection has been the standard method for decades but errors are frequent, storage costs are prohibitive, and the costs of double data entry are high. Electronic methods of data collection have been developed in order to merge the process of data collection and data entry [2]. Handheld devices such as personal digital assistants (PDAs) are increasingly being used instead of paper and pencil methods of data collection [1,3]. PDAs are not without problems of their own, however, including the challenges associated with having to download data (often to expensive laptops in the field). In addition, data can be corrupted if PDAs are damaged, or data can be lost if PDAs are misplaced or stolen.

Wireless and mobile phone technologies have the potential to overcome some of these limitations. Moreover, they can be adapted for use in field research settings. Low and middle-income countries lack the infrastructure in many research field settings to accommodate adequate fixed line internet access, whereas wireless networks allow access to telecommunications in a region where fixed lines remain limited. In Africa, mobile users account for 83 per cent of telephone subscribers, a higher proportion than any other region in the world [4]. South Africa leads the continent in mobile penetration with 36.4 mobile phones per 100 population [5]. Use of mobile phones is widespread even in remote areas of rural South Africa [6].

The use of mobile technology as a research instrument is in its infancy, however. Studies conducted in developed country settings have investigated the use of cell phones on the patient end to generate feedback for improved chronic illness care and monitoring [7-9], increased medication compliance [10] and smoking cessation [11], or reduced missed clinic visits [12,13]. Additionally, other studies have investigated the use of cell phones on the provider end to transmit images for documentation [14] or diagnostic purposes [15-18]. However, few studies have investigated the use of mobile phones as a data collection tool in low income countries. One demonstration project in Peru showed that a cell phone-based system could be used to collect real-time data on adverse events occurring during the course of a randomized trial [19]. The Millennium Villages Project has also begun efforts to use mobile phones to monitor livestock health, facilitate the timely transfer of patients to appropriate health facilities, and support community health workers in the field [20]. Finally, there are numerous anecdotal reports [21], but few published studies exist.

In the context of poor research infrastructure and of increasing demands for large scale health surveys, the affordability and availability of mobile phones and wireless networks make them a viable alternative to traditional paper and pencil methods and even PDAs. In this paper we report on the use of mobile phones in a survey conducted in a peri-urban settlement in South Africa using lay community health workers. The aim of this survey was to list and map households, and to collect selected socio-demographic data of household members. We investigated the feasibility, the ease of implementation, and the extent to which community health workers with little experience in electronic data collection could be trained and successfully supervised to collect data using mobile phones in a large health survey.

## Methods

### Setting

The site for the survey was Umlazi, a peri-urban settlement close to Durban in South Africa. Umlazi has a mixture of formal and informal housing. It is a relatively well resourced peri-urban area but with a non-optimally functioning health system. The infant mortality rate is around 60 per 1,000 live births [22], and, while there is no reliable figure of the neonatal mortality rate (NMR), most estimates place the NMR at about 25 per 1,000 live births. The HIV prevalence amongst antenatal clients in South Africa is 44% [22]. In order to check the homogeneity of clusters in preparation for a planned cluster randomized trial we conducted a baseline survey.

### Data collection and training

The data were collected by local women hired as community health workers (CHW's). Twenty-four clusters covering the proposed study area were selected prior to survey implementation. Every house in all 24 clusters was listed using the South African national census listing strategy [23]. The senior member of each household in each cluster was interviewed. All interviews were conducted face-to-face, in the homes of the study participants.

None of the 24 CHW's had any previous experience of data collection, but all had personal mobile phones and were proficient in the use of short message service (SMS) messaging. Training for the data collection protocol was conducted for all the CHW's over a two day period with the data quality control officer present. Training consisted of a general orientation to using the phone and its data collection software, accessing the questionnaires on the phone, and standard care of the device. The training also included information on troubleshooting and how to deal with technical difficulties that might arise with the mobile phones. Survey questions were pilot tested prior to implementation to ensure readability and to ensure that the questions were understandable to respondents. Standard interview protocols were used to direct how interviews were conducted, and standard operating procedures were developed.

### Mobile Researcher: the mobile phone data collection system

Implementation of the mobile system ("Mobile Researcher") was a partnership between the Health Systems Research Unit at the Medical Research Council of South Africa and a private digital solutions company, Clyral. The only hardware requirement for Mobile Researcher is that the mobile phone must be enabled for the Java programming language. The software consists of a Java 2 Micro Edition (J2ME) client which is installed on participating project staff handsets and communicates with the Mobile Researcher web application, also developed on the ASP .NET 3.5 framework. Java's core code is available under open-source distribution terms. We established a web-based system that allowed electronic surveys or questionnaires to be designed on a word processor, sent wirelessly to standard entry level mobile phones, and then used in interviews. Survey completion takes place offline, and no network coverage is necessary.

**Figure 1:**
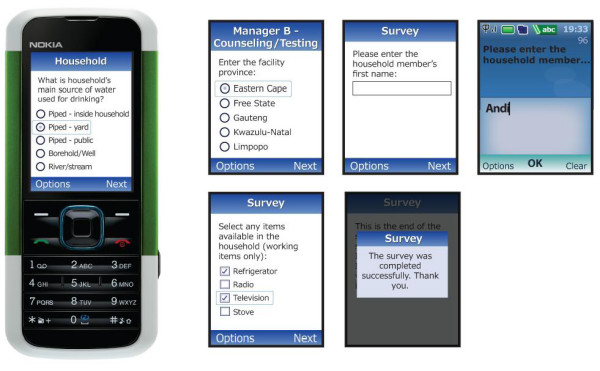
**Screen shots of survey on the mobile phone**.

Completed surveys are automatically uploaded to the host computer. If there is no mobile network coverage, completed surveys are stored securely until a signal is found at which time completed surveys are uploaded. Entry level mobile phones can store approximately 50 completed standard-length surveys. Mobile Researcher can incorporate multiple choice, free text, numeric, date, time and other question types (see Figure 1).

**Figure 2:**
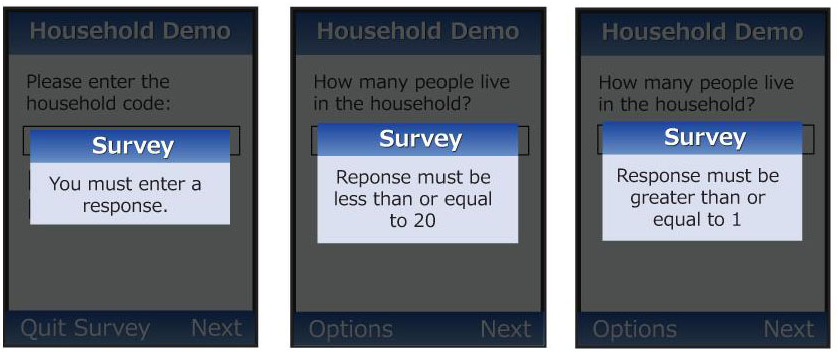
**Enforced validation**.

**Figure 3:**
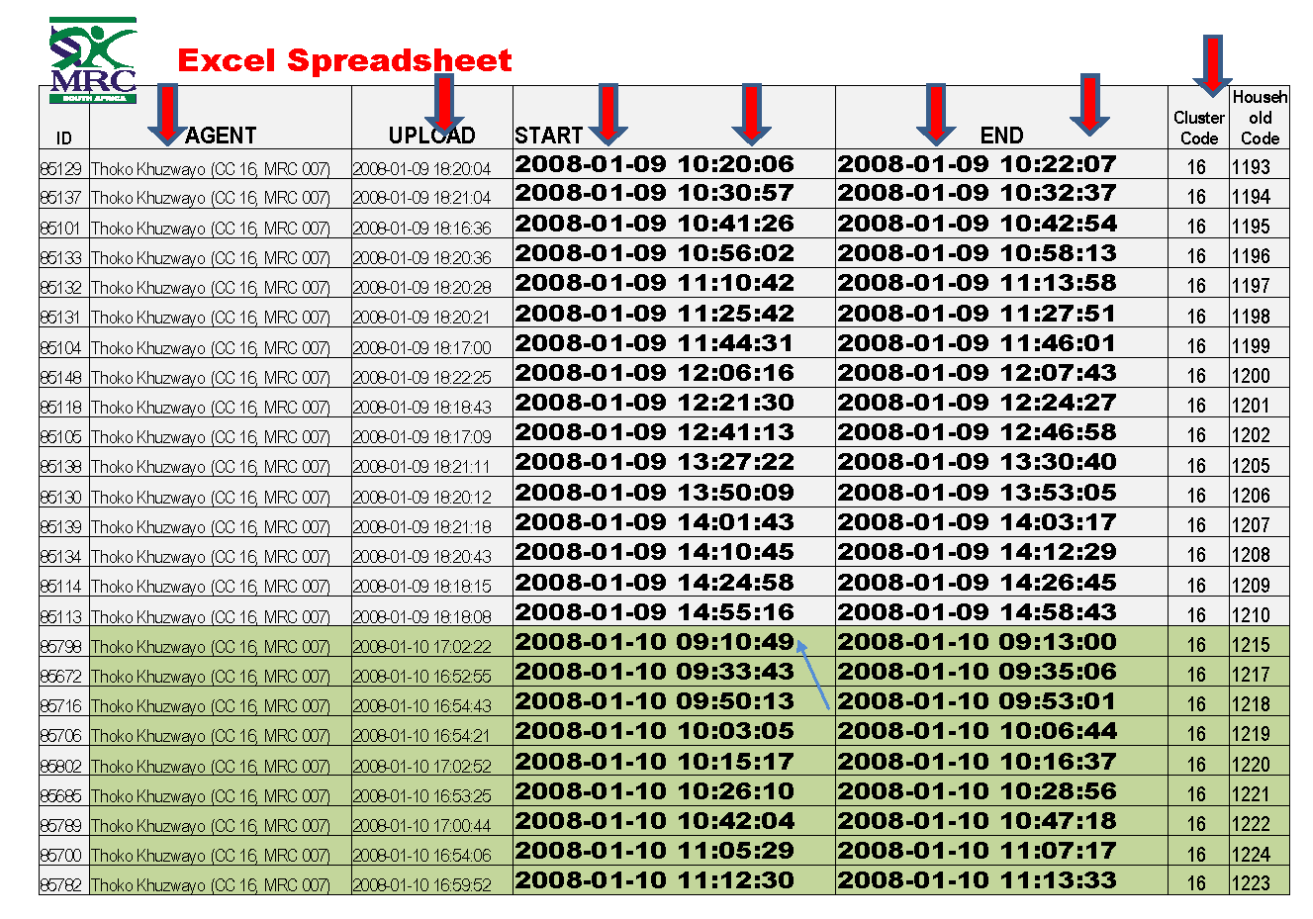
**Generated spreadsheet**.

In addition, Mobile Researcher can also accommodate branching and skip logic as well as enforced validation in the field (Figure 2). Survey data are uploaded using low cost general packet radio service (GPRS). A web-based interface was developed to facilitate the review and exporting of results in standard file formats such as comma separated values (CSV) and Microsoft Excel. Figure 3 is an illustration of an Excel report that provides formatted information files on data collector, time taken for the participant id and household code and when the actual upload of the data took place. Outputs such as this (Figure 3) can be generated as often as the investigator would like. Alternatively, built-in graphs and reports on the web-based interface permit real-time visualization of survey responses. Supervisors can communicate with data collectors directly, either through a call to the mobile phone or through SMS messaging. For logistic and supervisory purposes SMS messages can be sent through the web-interface to one or more data collectors at the same time.

All survey data were encrypted, thus maintaining the confidentiality of responses. Communication between the browser and the server was encrypted using 128-bit SSL. System servers were secured by firewalls to prevent unauthorised access and denial of service attacks, while data was protected from virus threats using NOD32 anti-virus technology. Access to the web-interface is protected by passwords. In the current study, access to the data was restricted to the principal investigator, the project manager, data quality officer and web administrator.

## Results

### Implementation of the Survey

Over the course of four months, 39,665 households were surveyed with no data loss. Quality checks were performed in real-time, and inconsistencies were detected, rectified, and cleaned in a timely manner. There were no hardware or software failures using the mobile phones. The automated graphs and reports allowed the project manager to visualize outputs such as survey completion count on an hourly or daily basis or the average survey completion time.

**Figure 4:**
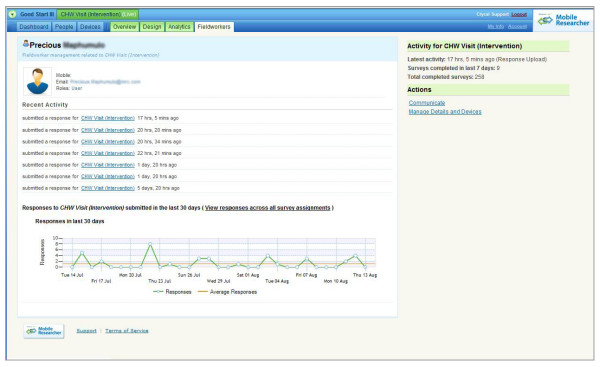
**Community health worker output**.

The mobile phone, together with the web-interface, allowed the project manager to monitor work rate, attendance (given that this was a community based survey), commencement of work, and cessation of work (see Figure 4). The automatic uploading of completed surveys provided ongoing data on interview start time, end time, and time taken to complete each survey (Figure 4). Regular meetings were held with Clyral to review the data collection process and address any difficulties.

One of the major advantages of the mobile phone data collection method was in the real-time detection of probable data falsification. Based on our experience with the CHW training sessions, and the short survey we expected that negotiating entry into the house, completion of the survey and the walk to the next house would require a total of 25 minutes. Using the web-based interface and the real-time monitoring of CHW output, we were able to detect an instance of data fabrication on the day that it occurred (see Figure 3 and Figure 4 for the data that is available to produce this information). Using this data, we observed that one CHW was submitting completed surveys every five to ten minutes. This was thought to be an impossibly short time given our expectations about the amount of time it would reasonably take to move from household to household, introduce the study, obtain informed consent, and complete the survey. The principal investigator was contacted, and a brief meeting was held with the CHW and it was established that she was completing surveys while sitting at home. Without revealing the identity of the CHW responsible, we also informed the rest of the CHW's about the incident in order to establish awareness among the staff of the monitoring potential of the supervision system. This was the only instance of data fabrication that we discovered. Rapid detection of fabricated data based on paper and pencil surveys can be difficult. For field research arrangements that involve weekly or monthly supervisory meetings, correction of the data fabrication may not occur until weeks to months after the fabrication has begun.

### Cost of Implementation

Given the minimal hardware requirements, we selected an entry level mobile phone (NOKIA 2626), which was priced at ZAR400 (US$40) in South Africa at the time of the survey. Surveys were billed per individual question completed. In this survey, the cost per completed survey was US $0.30 (with no data storage or data entry costs).

## Discussion

In a household survey, using previously untrained CHW's as data collectors, we utilized mobile phones to enter and upload data at the point of collection. The software application and web-based interface enhanced real-time supervision of data collectors. We were able to implement this survey with low direct costs of materials. While we are not able to compare costs to a paper based approach in this study, it is likely that a cost of $0.30 per survey compares favorably. Were we using a paper based approach this figure would include paper costs, printing costs as well as data entry costs. The issue of the cost effectiveness of the system needs to be explored in further research that employs a comparison group. Overall, our findings demonstrate that mobile phone based data collection is feasible at scale.

Real-time supervision of CHW performance was a significant advance over previous implementation work. It has been argued that pen and paper are prone to fabrication [21]. Our web-based interface permitted a previously impractical degree of detailed, hour-by-hour supervision, which markedly improved our ability to detect one type of data fabrication. Other types of data fabrication could still go undetected; for example, a data collector could key in random answers on a timed basis, and our supervisors would be unable to detect this activity. Such fabrication could only be detected and eliminated with the use of a more expensive mobile phone with GPS capabilities that would enable supervisors to track CHW movements. The web-based interface had other advantages as well. The automated graphs and real-time information allowed supervisors to focus their time on other aspects of quality control and solving logistical difficulties in the field.

With regards to the direct costs of materials, the automated uploading of completed surveys obviated the step of having to transfer data in the field from a PDA to a laptop. This is a significant cost saving, as laptops are often the most expensive up front cost in studies using PDAs for data collection [2]. In addition, the web-based interface permitted us to monitoring the costs of uploading survey data in real time.

Automated data upload from the mobile phone to the server significantly reduces data loss due to PDA damage, theft or loss because of the time elapsed between data collection and data upload. Prior studies have reported technical problems with data upload and download using PDA-based survey systems [24], as well as difficulties in remote sites with data upload due to electrical interference with telephone lines and switchboard difficulties [1]. In addition, there have been considerable problems noted with the instability of the "active synchronization" process when transferring data from PDA to computer [25]. In our study, there was no data loss. While this does not guarantee that data loss cannot occur with this system, we believe the chances of data loss were significantly minimized.

Our mobile phone based survey apparatus may be particularly suited for conducting survey research in rural areas. In surveys where multiple research sites may be remote and dispersed, and where vehicles have to be used to travel from site to site to download data onto laptops, the mobile phone based data collection system may be a significantly cheaper option. Importantly, survey storage (up to 50 completed surveys can be stored on an entry-level mobile phone) and delayed upload permits surveys to be conducted in areas where there is no mobile phone coverage.

Mobile phone based surveys have other advantages and disadvantages that may be particularly important to researchers depending on their needs and the study setting. The automatic uploading of surveys and encrypted access to the web-based interface contributes to improved data security and respondent confidentiality. In diverse populations where multiple languages are spoken, multilingual paper assessments are cumbersome and costly [26]. Mobile phones (like PDAs) easily integrate multiple language assessments with a simple drop down menu of language options. With regards to potential disadvantages, one of the drawbacks of using a paperless system is that there is no paper questionnaire to review in the event that problems are detected in the field. Also, although the automated data upload reduces the potential for data loss, mobile phones are valued items in resource-limited settings [27]. Thus mobile phones can be stolen, or may cause research staff to be targeted when conducting household surveys in high-crime areas. We chose to use entry level mobile phones for this very reason, but we did also permit the use of a paper and pencil version of the survey in isolated instances (for instance, if the CHW's felt threatened in a particular household or area). It is tempting with the introduction of a new technology to see it as a panacea for wide ranging methodological challenges that are common to most research. Using mobile phones for data collection will for instance not be able to address issues such as household access, selecting an appropriate sample that permit generalization of findings, or the complexities of clustered sampling to reflect population statistics. On the other hand, the immediate real time access to data significantly improves data quality, while the complex skip patterns of the mobile phone programme provides comprehensive validity and readability checks within the instrument.

## Limitations

Our findings should be considered with the following limitations in mind. First, it should be noted that our study design did not incorporate a control group. Thus, a randomized basis for comparison is missing. Prior research has made use of control groups to evaluate the use of PDA technology for data collection [21], so this could be incorporated into future research on mobile phone technology. In addition, controlled studies of mobile phone data entry error rates are needed [26]. Second, we employed a private digital technology company to provide programming support for the mobile phone software. The issue of proprietary versus open source software is a particularly important issue for mobile phone research in low and middle income countries. The Mobile Researcher software is not at present open source, but there are plans for it to be made open-source within the next six months. We would argue that, given budget constraints in most projects in low and middle income countries, open source software is important. We acknowledge however, that proprietary solutions may also be of value to research teams in specific instances.

## Conclusion

Our experience with a large scale baseline survey suggests that the real-time quality control and data collector supervision enabled by the use of a mobile phone based survey system make this an attractive management option and preferable to a paper based approach. This mobile solution has the potential to be scaled up in an extensive way for teams and studies of almost any size. The benefits of mobile technology, combined with the improvement that mobile phones offer over PDAs in terms of data loss and uploading difficulties, make mobile phones a feasible method of data collection that needs to be further explored. Rigorous controlled trials comparing data accuracy, readability, reliability and validity checks comparing paper based approaches, PDA's and mobile phones are needed. This should also include a cost effective analysis in small as well as large scale surveys.

## Competing interests

The authors declare that they have no competing interests.

## Authors' contributions

MT: Designed the study, designed the assessment instruments, drafted the first version of the paper. He has taken a major role in writing the submitted paper and approved the final version. WS: He has taken a major role in planning the study, writing the submitted paper and approved the final version. He has no other potential conflict of interest to declare.

YS: She has taken a major role in performing data quality of the study, writing the submitted paper and approved the final version. TD: She has taken a major role in planning the study, writing the submitted paper and approved the final version. MC: He has taken a major role in planning the study, writing the submitted paper and approved the final version. PJ: She has taken a major role in supervising the implementation of the study, writing the submitted paper and approved the final version. ACT: He has taken a major role in writing the submitted paper and approved the final version. DJ: She has taken a major role in writing the submitted paper and approved the final version.

## Pre-publication history

The pre-publication history for this paper can be accessed here:

http://www.biomedcentral.com/1472-6947/9/51/prepub
